# Attribution of mortality to the urban heat island during heatwaves in the West Midlands, UK

**DOI:** 10.1186/s12940-016-0100-9

**Published:** 2016-03-08

**Authors:** Clare Heaviside, Sotiris Vardoulakis, Xiao-Ming Cai

**Affiliations:** Environmental Change Department, Centre for Radiation, Chemical and Environmental Hazards, Public Health England, Oxon, UK; School of Geography, Earth and Environmental Science, University of Birmingham, Birmingham, UK

## Abstract

**Background:**

The Urban Heat Island (UHI) effect describes the phenomenon whereby cities are generally warmer than surrounding rural areas. Traditionally, temperature monitoring sites are placed outside of city centres, which means that point measurements do not always reflect the true air temperature of urban centres, and estimates of health impacts based on such data may under-estimate the impact of heat on public health. Climate change is likely to exacerbate heatwaves in future, but because climate projections do not usually include the UHI, health impacts may be further underestimated. These factors motivate a two-dimensional analysis of population weighted temperature across an urban area, for heat related health impact assessments, since populations are typically densest in urban centres, where ambient temperatures are highest and the UHI is most pronounced. We investigate the sensitivity of health impact estimates to the use of population weighting and the inclusion of urban temperatures in exposure data.

**Methods:**

We quantify the attribution of the UHI to heat related mortality in the West Midlands during the heatwave of August 2003 by comparing health impacts based on two modelled temperature simulations. The first simulation is based on detailed urban land use information and captures the extent of the UHI, whereas in the second simulation, urban land surfaces have been replaced by rural types.

**Results and conclusions:**

The results suggest that the UHI contributed around 50 % of the total heat-related mortality during the 2003 heatwave in the West Midlands. We also find that taking a geographical, rather than population-weighted, mean of temperature across the regions under-estimates the population exposure to temperatures by around 1 °C, roughly equivalent to a 20 % underestimation in mortality. We compare the mortality contribution of the UHI to impacts expected from a range of projected temperatures based on the UKCP09 Climate Projections. For a medium emissions scenario, a typical heatwave in 2080 could be responsible for an increase in mortality of around 3 times the rate in 2003 (278 vs. 90 deaths) when including changes in population, population weighting and the UHI effect in the West Midlands, and assuming no change in population adaptation to heat in future.

**Electronic supplementary material:**

The online version of this article (doi:10.1186/s12940-016-0100-9) contains supplementary material, which is available to authorized users.

## Background

High ambient temperatures can have negative impacts on health in terms of increased mortality or morbidity, and several studies have attempted to quantify heat-related health effects for the present day or for future years, using observations and climate change projections [1–6]. During prolonged periods of very hot weather (heatwaves), mortality can increase substantially, particularly amongst the elderly.

The severe European heatwave of 2003 had wide ranging impacts on public health, the economy, infrastructure, and ecosystem health [7]. The effects were particularly noticeable in northern and central Europe, where it has been estimated that up to 70,000 excess deaths were associated with the heatwave in August 2003 [8]. France was particularly affected, with an estimated 14,802 excess deaths reported, 2,085 of which were in the city of Paris [9], and high night time minimum temperatures were associated with increased mortality in the centre of Paris [10]. In England, the Office for National Statistics (ONS) estimated that 2,091 excess deaths were associated with the heatwave, of which, 616 were estimated for London [11]. The highly urbanised region of the West Midlands, the focus of this study, had an estimated 130 excess deaths during the heatwave period [11].

The Urban Heat Island (UHI) is a phenomenon whereby cities and towns exhibit higher temperatures than rural or suburban surroundings, due to a combination of factors including urban materials and morphology, anthropogenic heating and a lack of moisture in urban areas. The UHI intensity (generally defined as the difference in air temperature between built up urban areas and rural locations) in the urban canopy layer is more pronounced at night time, where it can reach values of up to 10 °C in large cities, such as Manchester [12] when conditions are favourable, for example, with clear skies and low winds. In Birmingham, the UHI intensity has been estimated to be up to 7 °C [13, 14]. This urban increment in temperature (compared with nearby rural temperatures) means that people who reside in built-up urban areas are likely to be exposed to higher temperatures than those residing in rural areas. The complex relationship between the built form, local temperatures and health has been studied by developers and planners, for example, in cities in the UK and Australia, particularly London and Melbourne [15–18]. The influence that the UHI has on indoor temperatures is difficult to quantify, and requires greater understanding, as people spend the majority of their time indoors and the UHI can exacerbate building overheating during heatwaves, in particular, reducing cooling rates at night [18, 19].

Since the frequency of heatwaves is likely to increase in future, and it has even been estimated that events as severe as 2003 may become as frequent as once every 2 years by 2040 [20–22], it is likely that with constant or increasing urbanisation, the associated health risks will continue to be a cause for concern for the UK public health sector in future decades, and quantification of potential impacts is required.

Calculations of the potential health effects of heatwaves can be based on a variety of methods. For past or current heatwave events, temperature exposure is often based on observations from stations which are not always sited within city centres. Estimates of future health burdens often make use of modelled temperature from global climate models. Due to computational efficiency requirements, this global output is often at too coarse a resolution to resolve features at city scales. Since urban land surfaces are not represented in many global climate models, climate projections often neglect the added effect that towns and cities have on local temperatures, including the UHI effect. This means that health impact assessments based on climate projections may underestimate the potential health impacts of very high temperatures in cities during heatwaves, compared with models which include urban temperature effects.

This study attempts to address methodological shortcomings in estimating heat-related health impacts in an urban area by using simulated temperature data to characterise population exposure. For example, sparse networks of temperature monitoring stations based away from the most populated areas may not best represent the temperature which is typically experienced by the majority of the population residing close to the centre of cities. The use of high resolution, gridded temperatures based on model simulations which cover all urban and rural areas within a region, and the application of population weighting, help to address these limitations.

Previous work has used modelled temperature on a 1 km grid for London to estimate UHI impacts on health by summing health effects by UHI zones depending on location [23] and investigated how the UHI may change in future, based on urban parameterizations added to global simulations from the HadAM3 model [24]. To our knowledge, the present study is the first to attempt to quantify the heat related mortality for a specific event, and then to calculate the proportion of this mortality which can be attributed to the UHI effect by using simulations of urban and rural scenarios. We present quantitative estimates of the risks to health associated with increased temperatures due to a) the UHI and b) projected climate change, using the heatwave of 2003 and the region of the West Midlands as a case study, assuming that population adaptation to heat does not change in future. The use of high resolution simulations improves on the use of the limited number of fixed measurements by revealing spatial variations in temperature across the region.. As well as estimating the contribution of the UHI to mortality during the 2003 heatwave, we calculate the potential impacts on health of an increase in temperature such as we might expect in future decades under climate change scenarios, in addition to the UHI intensity. This not only allows us to anticipate future health burdens, but also acts as a useful comparator to the estimated health impacts attributed to the UHI.

## Methods and data

### Temperature modelling

**Fig. 1:**
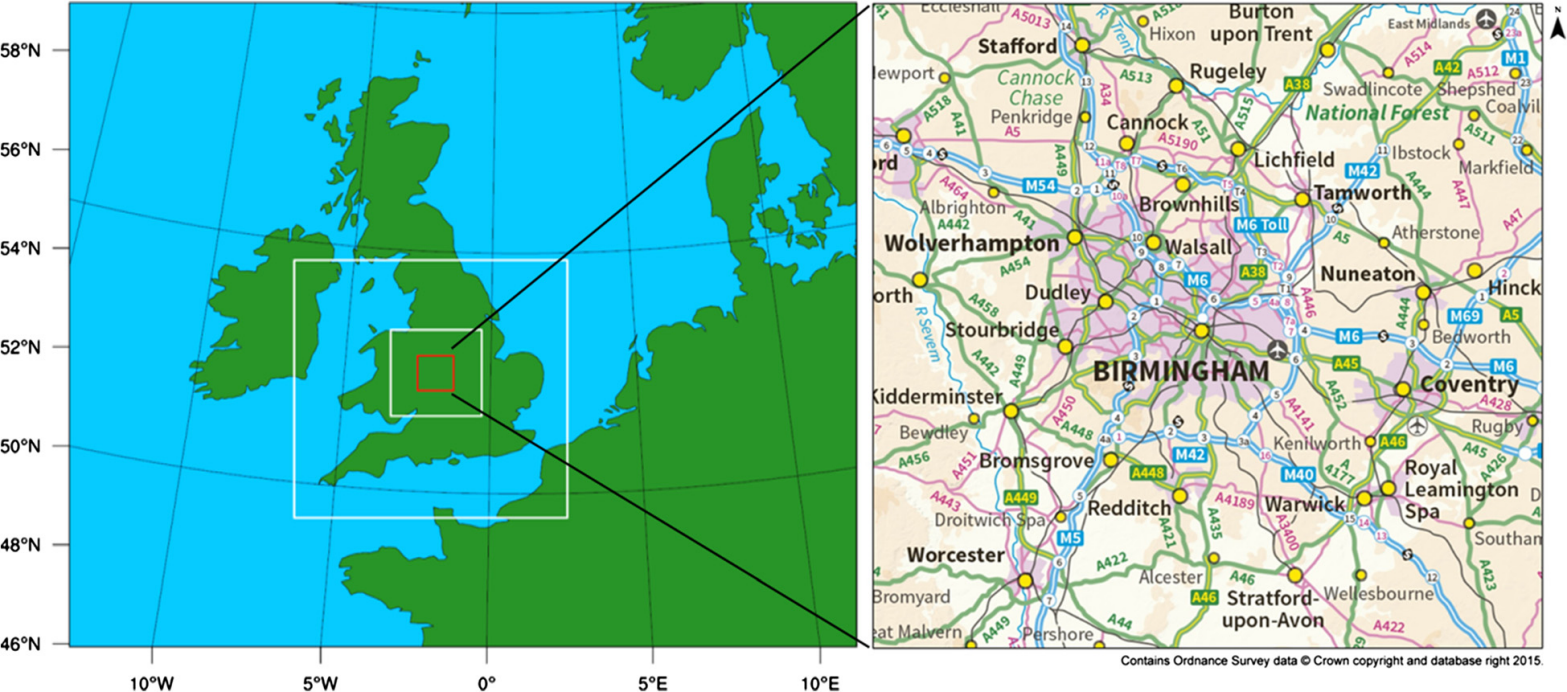
Geographic area covered by the WRF model simulations. Model resolution increases as boxed domains decrease in size, and is 1 km^2^ in the central domain (red box) which covers the West Midlands region study domain (right), which is approximately 81 km by 78 km

Within the West Midlands, temperature observations are relatively sparse, with only 4 or 5 station records of air temperature available during the heatwave of 2003. Due to the high degree of spatial heterogeneity in land use across the West Midlands, limited point measurements of temperature do not fully represent temperature variations across the region. To address this limitation and in order to assess heat related health impacts, we have used modelled, gridded air temperature at a horizontal resolution of 1 km x 1 km and a height of 2 m, covering an area of ~6,300 m^2^ within the West Midlands region (Fig. 1). We used the WRF (Weather Research Forecasting) model [25] to simulate hourly temperatures throughout the heatwave period, from midnight on the 1^st^ August until midnight on the 10^th^ August 2003. The hourly temperatures output from WRF were averaged to give daily (24 h) mean temperature for the Health Impact Assessment. This set up of the WRF model, with an urban canopy scheme (BEP–the Building Energy Parameterization scheme) set up specifically for Birmingham and the West Midlands was previously validated against all relevant and available observations obtained from a mixture of rural, semi-urban and urban UK Met Office (MIDAS) weather stations (downloaded from BADC: www.badc.nerc.ac.uk/home/index.html) to ensure satisfactory model performance [13]. The use of the multilayer BEP surface urban canyon scheme with 3 separate urban land classifications, adapted from [26], allows us to represent typical features of urban areas of the West Midlands. Previous model runs using WRF with BEP showed that the UHI intensity was about 3 °C on average, throughout the heatwave period, and reached a maximum of 7 °C at 2 am on the 6^th^ August. Full details of the model configuration and validation, the three urban land categories used, and estimates of the intensity of the West Midlands UHI can be found in [13].

To investigate the effect that urban surfaces have on local temperature, and the associated health impacts, we compared 2 different WRF model simulations. The first simulation (named ‘urban’) was run to accurately capture the local temperature throughout the August 2003 heatwave in the West Midlands and is based on specifically adapted information on land use category across the modelled domain, including a breakdown of urban areas into 3 separate categories (commercial, high-intensity residential and low-intensity residential). The second simulation (named ‘rural’) was run as a theoretical simulation, for the same region and the same time period as the ‘urban’ run, but with all urban land surfaces (about 40 % of the total land cover within the modelled domain) replaced by a representative rural category (in this case ‘dryland cropland and pasture’) to characterise a rural landscape [13]. Since both simulations are run for the same time period and over the same geographical area, we can attribute differences in temperature between the urban and rural simulations to land use, which allows us to quantify the heat related health effects of the UHI in the West Midlands. We define the UHI intensity in this study to be the difference in temperature between the ‘urban’ and ‘rural’ simulations, as previously defined in [13].

### Climate projections

The UK Climate Impacts Programme provides the most recent climate projections at a resolution of 25 km^2^ specifically for the UK (http://www.ukcip.org.uk/). The most recent set of projections (UKCP09) [27] date from 2009 and provide probabilistic future UK climate projections up to the 2080s, based on 3 possible emissions scenarios: high, medium and low, which refer to the IPCC (Intergovernmental Panel on Climate Change) Special Report on Emissions Scenarios (SRES) of A1FI, A1B and B1, respectively [28]. The UKCP09 climate projections are available as monthly mean values, and urban areas are not fully represented in the model since they are generally too small compared with the climate model resolution. This means that these climate projections can only give an indication of monthly mean temperature changes in future, and they give no information about the UHI effect.

Our method to estimate future temperatures during heatwaves in urban areas therefore involves adding the incremental temperature from the monthly mean climate projections uniformly to the high resolution urban temperature simulations. This technique combines projections of a future change in mean summer temperature with the modelled UHI effect experienced during an extreme heatwave, assuming no changes in urban density and morphology from the 2003 model configuration.

To investigate the potential impacts of climate change on mortality in the West Midlands, we have taken projections for the West Midlands for the change in daily mean temperature in August for a medium emissions scenario for a range of future decades (the 2020s, 2050s and 2080s), expressed as a single value for each decade. We added the change in temperature from the baseline period to the modelled urban temperatures during the heatwave of 2003 from WRF to give a range of potential daily mean temperatures to be used as input for the Health Impact Assessment.

### Demographic data

A gridded dataset of residential population for 2003 at 100 m x 100 m resolution for the modelled domain was extracted from the UK National Population Database, version 2 (NPD2) [29] and aggregated to each of the WRF modelled 1 km x 1 km grid cells. Population weighted mean temperature for the population within the modelled domain in the West Midlands was calculated by multiplying the total resident population in each 1 km cell by the temperature in each 1 km model cell, summing the values for all grid cells within the region, and dividing by the total regional population. This population weighted temperature was then used in the health impact assessment to calculate health burdens from heat during the heatwave of 2003.

Daily mortality counts for the West Midlands were based on aggregated data obtained from the ONS during the period 1993–2006 for the West Midlands region. All-cause mortality including external causes in the West Midlands for the 1^st^–10^th^ August 2003 was used for the health impact assessment.

For the health impact assessment for future years based on climate projections, changes in population in the West Midlands for the 2020s were taken into account by using population projections for 2025 from the Office for National Statistics (www.statistics.gov.uk) and adjusting baseline mortality rates accordingly. Regional population projections were only available up to 2037, so regional population estimates for the 2050s and 2080s were based on the available UK estimates for 2055 and 2085. A quadratic relationship between the West Midlands and UK population and between the years 2013 and 2037 was extrapolated out to 2085 to provide estimates for the West Midlands as a proportion of the UK total. It should be noted that the geographical coverage of the modelling domain does not cover the entire West Midlands (the modelled domain includes a population of 4.75 million, around 90 % of the total population of the West Midlands region, which was 5.31 million in 2003).

### Health impact assessment methodology

The heatwave of 2003 was associated with 2,091 excess deaths in England, according to the recorded mortality figures for August 2003, when compared with the expected baseline numbers calculated from the August average for 1998–2002 [11]. In the West Midlands, the number of excess deaths over the same period was estimated at 130. These figures are based on the difference between recorded and expected deaths for each region.

In the present Health Impact Assessment (HIA) an increase in temperature is statistically related to an incremental increase in mortality, taking into account daily variability in both parameters and potential confounding factors. The same method can then be applied to different scenarios with varying temperature exposure data (e.g. using the “urban” or ‘rural’ simulation), and the resulting health impacts calculated. This gives us an indication of the number of deaths we might expect based on particular exposure data, in this case air temperature. The number of heat related deaths (*M*) in the West Midlands region for the heatwave period, was estimated using the following relationship

1 $$ M={\displaystyle {\sum}_{i=1}^ND{M}_i\left(1-{e}^{\left(-b\varDelta {T}_i\right)}\right)} $$

where *N* is the number of days in the heatwave period, in this case *N* = 10, *DM* is the recorded daily mortality in the region, or baseline mortality rate, *b* is the slope of the exposure-response relationship for heat and mortality in the West Midlands and ∆*T*_*i*_ is the temperature on day *i* above a threshold for the West Midlands. The values of the slope of the exposure-response relationship, *b* and the threshold were originally derived by Hajat et al. [1], for each separate UK region, including the West Midlands, based on epidemiological analyses over the period from 1993 to 2006; the threshold temperature is set at 17.7 °C, which represents the 93^rd^ centile mean temperature for the West Midlands.

We apply Equation (1) to calculate daily mortality for the West Midlands from 1^st^ to 10^th^ August 2003, inclusive. The baseline daily mortality rate is based on figures for the West Midlands Government region for the heatwave period, and equates to an average of 147 deaths per day. *ΔT*_*i*_ is calculated using the difference between the population weighted daily mean temperature and the threshold value of 17.7 °C; the Relative Risk (RR, where *RR* = *e*^*(b∆T)*^) of mortality for heat in the West Midlands per 1 °C increase in temperature is 1.023 (95 % CI 1.016 to 1.031). The threshold and coefficient were derived using time-series regressional analysis to assess acute relationships between daily temperature fluctuations and mortality, assuming Poisson variation with scale overdispersion over summer months and with control for seasonal patterns in mortality. Relative humidity and day of week effects were adjusted for, although air pollution was not [1]. Temperatures on each day were calculated as an average of the present day and the previous day (apart from the first day) to allow for possible lag effects, and temperature on the final day (10^th^ August) was taken as that of the previous day as an approximation, since the simulation finished at midnight on the 10^th^, but health impacts may have continued due to the lag effect. This gives a total period of 10 days (1^st^–10^th^ August inclusive) for the health impact assessment. It is possible to calculate the potential impact of the UHI on health, by comparing the HIA for the ‘urban’ model run with the ‘rural’ model run. In the theoretical ‘rural’ case, all urban surfaces have been removed from the model, so that temperatures across the West Midlands do not include the UHI effect. The methodology for population weighting is applied in the same way to the ‘rural’ model run to allow for comparison with the ‘urban’ run.

When projecting future health impacts based on UKCP09 temperature projections, we applied the same exposure-response relationship as we did when calculating health impacts for the 2003 heatwave, which assumes no changes in population vulnerability in future.

## Results

### Population-weighted mean temperature in the West Midlands

We used high resolution, gridded temperature data in combination with gridded demographic information in order to accurately characterise population exposure to heat in the West Midlands region. This methodology addresses the limitations of estimates which rely on sparse point measurements of temperature, or which use geographical mean temperature over a region and do not take population weighting into account.

**Fig. 2:**
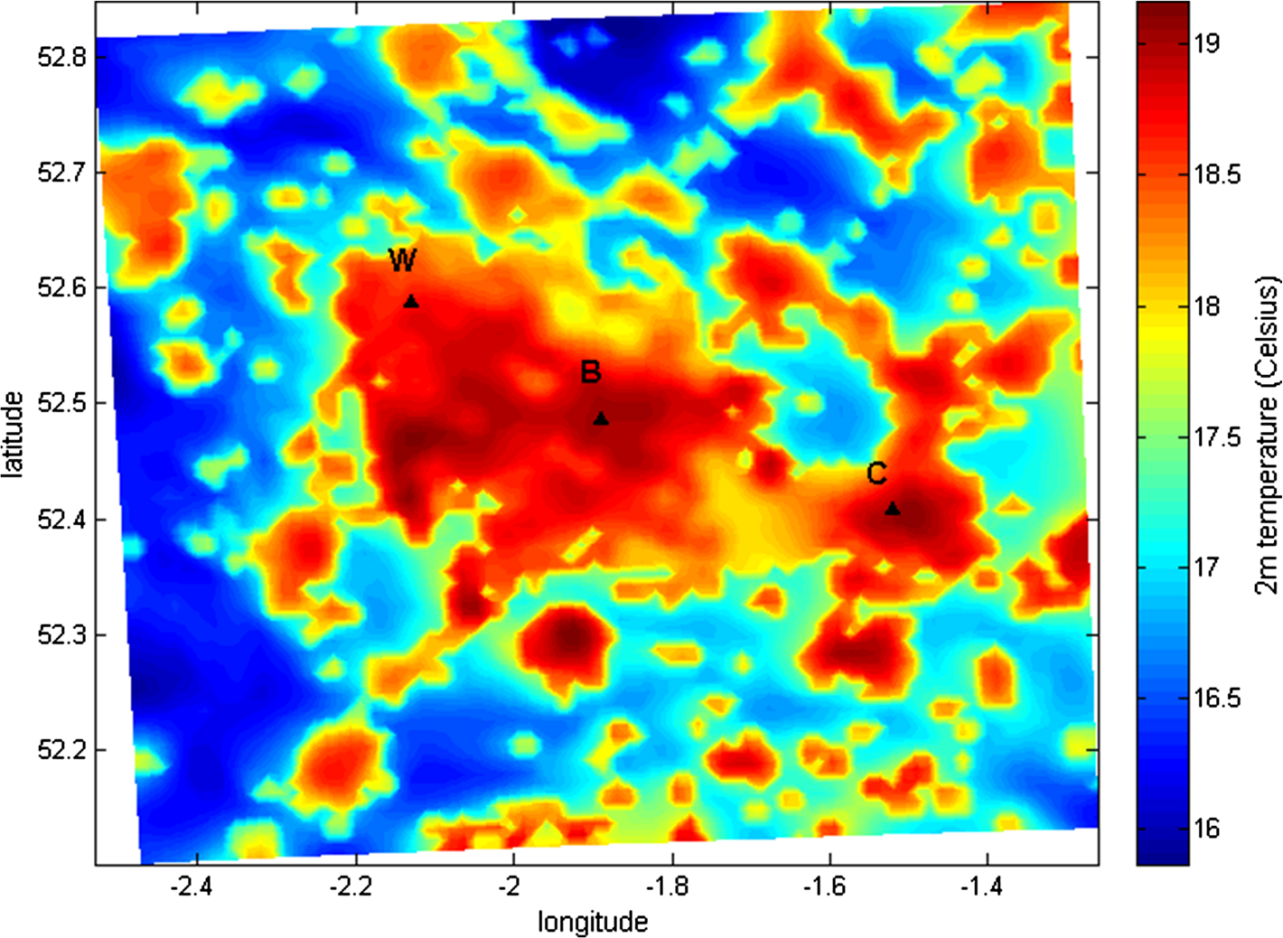
Mean hourly air temperature at height of 2 m for all hours from 1^nd^–10^th^ August 2003 for the ‘urban’ WRF simulation. Markers labelled W, B and C represent the cities of Wolverhampton, Birmingham and Coventry

Figure 2 shows the mean air temperature at 2 m above ground, simulated by the WRF model ‘urban’ run over the period 1^st^ -10^th^ August 2003. The higher temperatures are mostly found in Birmingham, in the centre of the domain, although other cities such as Wolverhampton and Coventry also exhibit higher temperatures than rural surroundings, so that on average, urban areas are around 3 °C warmer than rural areas. The average temperature across the whole of the modelled domain over the period from 1^st^ to 10^th^ August is 19.9 °C. If we only include night-time hours then the average temperature is 17.7 °C. These values represent a geographical mean temperature (Table 1). The highest modelled hourly temperature for Birmingham Centre was 31.7 °C on the afternoon of the 6^th^ August. Although temperatures are generally higher in daytime compared with night, the UHI intensity is most pronounced at night, meaning that there is generally a smaller diurnal temperature range in urban areas. This is largely due to the fact that urban materials slowly release heat that has been stored during the day into the atmosphere at night, to a greater extent than rural land surfaces. Heatwaves of durations lasting several days can therefore result in little relief from high temperatures at night in urban areas.

**Table 1:** Geographical mean and population weighted mean temperatures for the modelled domain for the period 1^st^–10^th^ August 2003

	Whole period	Night time only
Geographical mean temperature (*T*_*g*_)	19.9 °C	17.7 °C
Population weighted mean temperature (*T*_*pw*_)	20.7 °C	18.6 °C
Difference (*T*_*pw*_–*T*_*g*_)	0.8 °C	0.9 °C

**Fig. 3:**
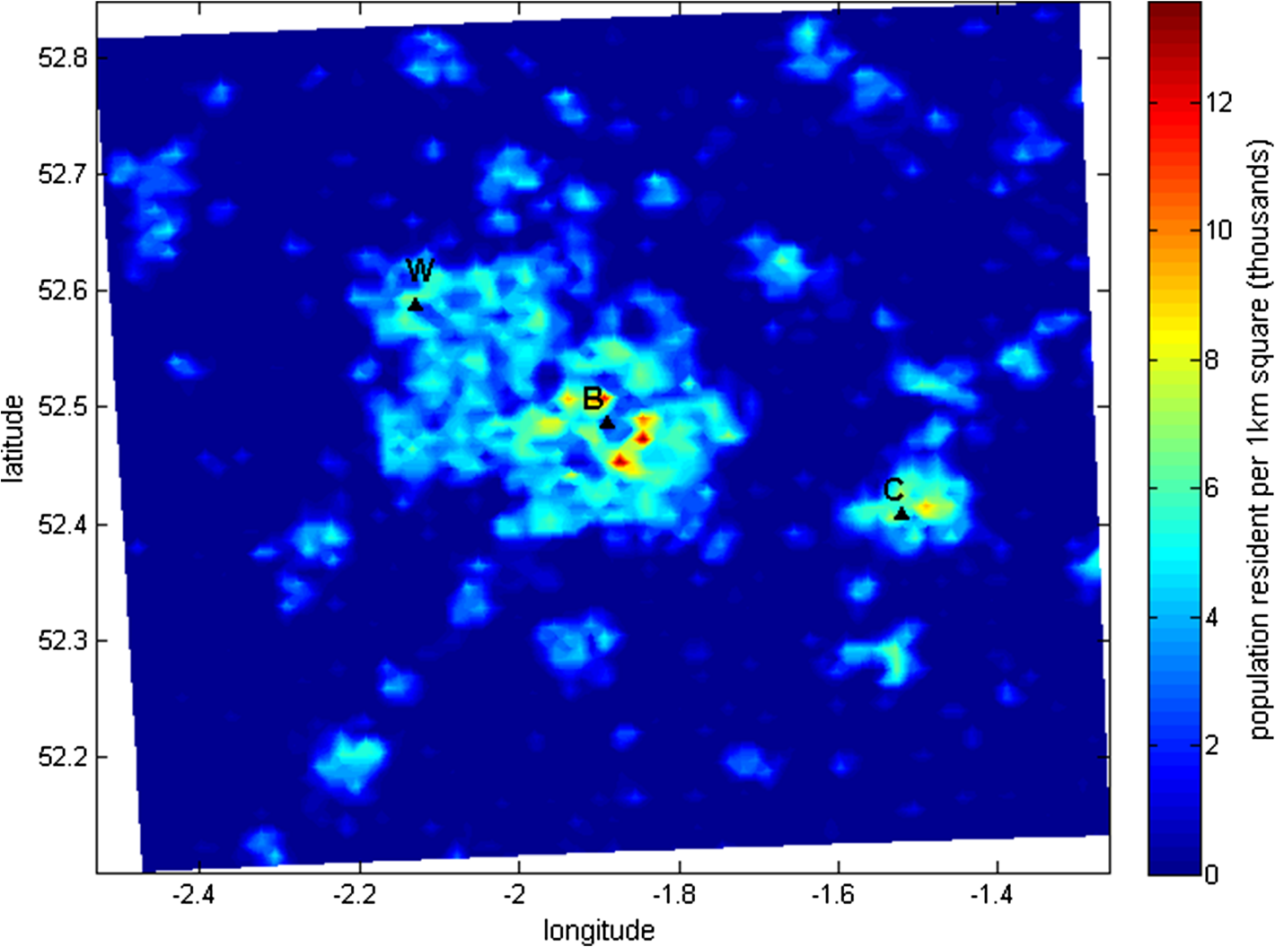
Total population per 1 km grid cell across the West Midlands modelled domain in thousands. City markers as in Fig. 2

Figure 3 shows the summed population resident within each 1x1 km grid cell of the modelled domain in the West Midlands. The spatial pattern closely mirrors the temperature pattern illustrated in Fig. 2, since the most highly populated areas are the most urbanised, and hence temperatures are generally highest in city centres due to the UHI effect. Population-weighted mean temperature exposure across the domain is given in Table 1.

The difference between geographical mean temperature across the entire domain and population weighted mean temperature based on the 1 km grid is around 1 °C in this case (Table 1). This indicates that neglecting population weighting could result in an under-estimation of the actual temperature exposure for people in urban environments, which is likely to have an effect on the results of health impact assessments based on temperature.

### Health impact assessment–heat related mortality during the heatwave

Following the HIA method, the estimated number of deaths during the 10 day heatwave period for the West Midlands was approximately 90, based on population weighted daily mean simulated temperature for Birmingham and the West Midlands, which includes the UHI effect. The results of the HIA using a geographical mean daily temperature give an estimate of only 73 heat related deaths over the same period. This implies that the underestimation of population exposure to heat resulting from using geographic mean rather than population weighted mean, of around 1 °C over the period, is the equivalent to an approximately 19 % lower estimation of mortality, in impact assessment terms. In absolute terms, we estimate that the mortality figure for this 10 day period would be underestimated by around 17 deaths by neglecting to use population weighted temperature. Although relatively small over a short period, this value will become more important for HIAs taking place over longer periods of time.

### The impact of the UHI on heat-related mortality

The HIA for the ‘rural’ simulation over the same heatwave period gives an estimated mortality of 43. The application of this theoretical simulation is a useful technique to investigate the influence that the UHI has on mortality, especially since climate models used for projections are usually based on rural land surface types that do not include UHI effects. For the 2003 case, the inclusion of the UHI intensity (the ‘urban’ simulation) more than doubles the predicted mortality from 43 to 90, meaning that by neglecting the local enhancement of temperature due to urban surfaces, we may only count around 48 % of the total mortality expected when using urban surfaces (Table 2). In other words, these calculations indicate that the UHI effect and the increased density of the population residing in city centres may account for 52 % of the total heat related mortality.

**Table 2:** Heat related mortality for the 2003 heatwave in the West Midlands based on a range of temperature metrics

Temperature metric used	Total estimated mortality for 10 day period	Percentage of pop. weighted estimate
Population weighted daily mean temperature (‘Urban’ run)	90	100 %
Geographical daily mean temperature (‘Urban’ run)	73	81 %
^a^No UHI case (‘Rural’ run)	43	47 %

^a^This is a theoretical calculation of mortality burden based on a ‘rural’ temperature simulation. The mortality attributed to the UHI intensity can be estimated from the difference in mortality between the ‘rural’ and ‘urban’ runs

### Health impact assessment–mortality for projected changes in climate

We estimate that the UHI effect could be accountable for around 52 % of the total heat-related mortality in the West Midlands during the 2003 heatwave. This is due to the enhanced temperatures simulated in urban centres when compared with rural surroundings. In the West Midlands the temperature difference between urban and rural model simulations over the heatwave period was on average 3.2 °C and reached a maximum difference of 5.6 °C [13]. This urban increment in daily mean temperature is large enough to significantly affect attributable mortality estimates (90 deaths with the UHI included, and 43 without).

Annual mean temperatures are projected to rise in future in the UK due to climate change from a range of around 2 to 5 °C by 2080, depending on location in the UK, based on a medium emissions scenario [27]. In the West Midlands, projections of increases in annual mean temperature range from 2.8 °C for a low emissions scenario and 4.7 °C for a high emissions scenario by the 2080s. Since the temperature increment due to the UHI in the West Midlands is of a similar size to projected temperature changes up to the 2080s, we provide estimates based on potential changes in temperature related to future climate change, and include the urban increment as modelled by WRF, although we acknowledge that changes in demographic, social and other factors by the 2080s will limit the reliability of the health impact estimations this far into the future. The UKCP09 projections (http://ukclimateprojections.metoffice.gov.uk/) suggest that UK summer mean temperatures are expected to increase more than winter mean temperatures. For a medium emissions scenario for the 2080s, winter minimum temperatures are projected to increase by up to 4 °C (less in the north of the UK) whereas summer mean maximum temperature are projected to increase by up to 6 °C. As well as a change in mean temperatures, research has shown that heatwaves are likely to become more common in future. Analysis of the 2003 European heatwave suggests that heatwaves of this scale are likely to occur every two years by the year 2040 [20–22].

The surface scheme used in the UKCP09 regional climate model does not include the UHI effect, and heat storage and release by urban materials is not modelled. This is because most urban areas are small compared to the model grid size (an exception being London). This means that the UKCP09 temperature projections should be considered, effectively, in addition to the UHI effect. We estimate future temperatures in the West Midlands by adding UKCP09 projected changes in temperature to the separately modelled urban temperature simulations. We have included a range of temperature projections from the UKCP09 climate projections, which are available as monthly means, in order to quantify the potential health burden due to heat for future heatwave conditions. We use projected changes in mean temperature rather than, for example, the temperature of the warmest day in summer, since we are using the already anomalously warm period of August 2003. The incremental temperature used here reflects the projected increase in daily mean temperature for August in the West Midlands region, for the 2020s, 2050s and 2080s under a medium emissions scenario, which corresponds to 1.4 °C, 2.7 °C and 4.0 °C, respectively. The use of monthly mean temperature changes for the West Midlands does not account for potential future changes in temperature variability on smaller scales, and this could be considered as a conservative estimate of future temperature, although this is partly accounted for by the inclusion of the extreme temperature event of 2003.

**Fig. 4:**
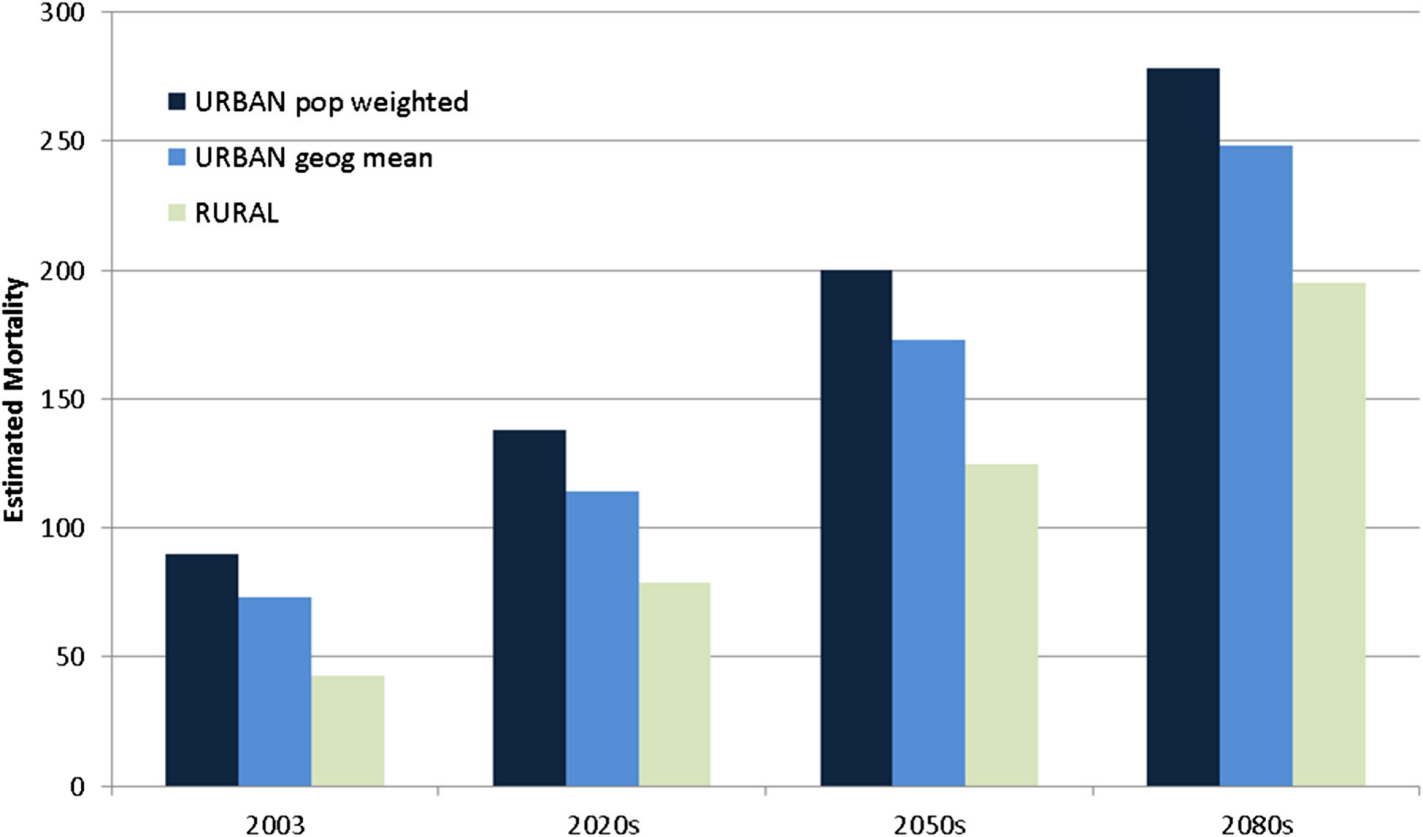
Estimated mortality based on health impact assessment for the heatwave of 2003, and for potentially similar heatwave events projected for 3 future decades, under a medium climate scenario. Dark blue bars (left) use population weighting and the ‘urban’ temperature simulations; light blue bars (middle) use the ‘urban’ temperature simulation but do not include population weighting, and light green bars (right) use the ‘rural’ temperature simulations

Figure 4 and Table 3 show estimates of mortality based on climate projections for future decades, compared with the August 2003 mortality estimates. Numbers in brackets indicate mortality based on a fixed population at 2003 levels (Table 3). Again, a number of temperature metrics have been used, so that mortality estimates are based on the urban model run (with both population weighted and geographical mean temperature) and the rural model run. Mortality estimates for future decades, excluding the UHI effect are around a third lower than those which include the UHI. When a change in population for future decades is considered, mortality estimates for a heatwave event similar to that of 2003, rise by 53 % in the 2020s, 122 % in the 2050s and 209 % by the 2080s from the baseline of 90 deaths in 2003. These estimates assume no adaptation to heat, since the same RR relationship has been used as for the calculations for 2003, (based on the original epidemiological study covering 1993–2006) and assume no changes to the UHI due to changes in weather, urban population density, rural conditions or urban development. They are also based on a medium emissions scenario, and future temperatures may vary from those projected here.

**Table 3:** Estimated mortality for the heatwave of 2003 and a range of future climate projections under a medium emissions scenario, based on 3 different modelled temperature metrics

	‘Current’ climate	Future climate projections (without population changes)		
Temperature metric	2003 heatwave	2020s	2050s	2080s
‘Urban’ population weighted	90	138(125)	200(159)	278(192)
‘Urban’ geographical mean	73	114(104)	173(138)	248(172)
‘Rural’	43	79(72)	125(101)	195(135)

## Discussion and Conclusions

We have used high resolution modelled temperature data across the densely urbanised and populated region of the West Midlands to estimate mortality associated with the heatwave of 2003. We found that a health impact assessment using population weighted temperature, rather than geographical mean temperature, increased the mortality estimates by approximately 19 %, since population weighted temperature was around 1 °C higher than geographical mean temperature. We used simulated temperatures from an ‘urban’ and a ‘rural’ model run for the West Midlands, which showed that the UHI intensity was on average around 3.2 °C throughout the heatwave period. We estimate there were 90 heat related deaths in the West Midlands during the heatwave of August 2003, using the ‘urban’ simulation temperatures, and only 43 using the ‘rural’ simulation temperatures. Based on these simulations, we conclude that the UHI effect contributes around half (52 %) of the heat related mortality over the heatwave period from 1^st^–10^th^ August 2003 in this region.

These results indicate that by excluding the UHI effect from temperatures used for HIAs, the full health impacts from heat during heatwaves similar to the August 2003 event are likely to be under-estimated in the West Midlands, by around 52 %. The population of Birmingham City is around 1 million, which makes it potentially comparable with many cities in Europe which are of a similar size or urban density. It is therefore likely that the UHI also affects mortality to a similar extent in many other cities, since it has been suggested that the UHI intensity is, broadly speaking, related to the logarithmic scale of city size [30], although research also suggests that urban planning initiatives which include urban greening and urban building modifications can reduce UHI intensity, so size should not be considered to be the sole determinant of the magnitude of the UHI effect, and urban morphology and characteristics are major factors [31–34].

Climate projections indicate there will be higher temperatures and more heatwaves in the UK in future, which is likely to increase the health burden from heat in urban areas. Climate projections which do not include urban temperature effects may underestimate the scale of future temperature increases. We have addressed this limitation by adding the high resolution urban temperature simulations to monthly mean climate projections to estimate temperatures in the 2020s, 2050s and 2080s based on a medium emissions scenario. Since heatwaves of the severity of the 2003 event are expected to occur more frequently in future [20], the technique of combining temperature fields can give a more realistic estimate of potential local temperatures than climate projections alone. When including projected growth in UK population, the HIA which includes population weighting and the UHI intensity, indicates increases in mortality by 53 % to 209 % from the 2020s to the 2080s compared with 2003 (heat related mortality estimates of 138 and 278 compared with the 2003 figure of 90) for a 2003 type heatwave in the West Midlands. Without the inclusion of urban temperatures, these figures are around 30–40 % lower.

These results have practical applications in terms of public health protection, and interventions such as the national Heatwave Plan for England, when considering that temperature exposure in urban areas may be a few degrees higher than existing predictions based on observations taken outside of cities. The position of vulnerable populations in urban centres can be more easily identified using high resolution temperature simulations, and we may expect these populations to be particularly exposed to the effects of the UHI in terms of ambient outdoor temperature. However, as well as outdoor temperature, there are many other factors which are likely to exacerbate health impacts in cities and which are linked to the UHI, such as indoor temperatures of dwellings, use of ventilation, passive and active cooling systems and the location of inhabitants within the hottest parts of buildings, such as upper floors.

The results presented here attempt to characterize the heat related health impacts of the 2003 heatwave in a large European city; however when projecting into the future, a number of assumptions have had to be made. We have used a set of climate change projections for the West Midlands based on a medium emissions scenario. However, there is large uncertainty in the scale of climate change we may experience, and which climate change emissions scenario is most likely to be realised in the coming decades. We do not attempt to predict future heatwave conditions or characteristics, so the methodology employed here includes adding an incremental mean temperature to the modelled ‘urban’ simulation. We also assume no population adaptation to heat in future, by assuming that mortality occurs at the same rate and above the same temperature threshold as it does currently. This may not be the case, if populations are expected to adapt to increased temperatures in future, although this is difficult to predict, quantify and account for in a HIA. Previous attempts to model adaptation to heat e.g. [5] have involved a variety of methods such as investigating variations in historical temperature-mortality relationships for the same region, or comparing locations with similar climates. Changes in the minimum mortality threshold temperature were also investigated as an indication of adaptation, and future effects were estimated, including the impact of various modifiers such as use of air conditioning and changes in urban factors. Adaptation, or acclimatization, was modelled in New York by using heat exposure-response relationships derived from cities with present day climates similar to those projected to 2050 in New York [6]. Recent reviews cover climate change and temperature related health impacts including adaptation to temperature effects, for heat and cold effects [4, 35, 36].

We have added future population figures based on ONS projections, but we cannot account for changes in population health in future, which may be related to improvements in healthcare, economic factors, medical research or changes in the age structure of the population. We also assume that the urban density and morphology remain as at the present day. The time period studied is relatively short, although the largest health impacts are thought to have occurred within this period and the severity of the heatwave was considered exceptional. We have not considered urban air pollution and its potential role in terms of mortality during the heatwave of 2003. It has been estimated that air pollution may have been responsible for between 18 % and 42 % of the total mortality in the UK during the heatwave of 2003 [37], although this estimate may include some double counting and it is difficult to isolate air pollution related deaths from heat related deaths. However, our lower estimate of 90 deaths compared with the estimate of 130 given by Johnson et al. [11] may be because the latter estimate includes excess deaths potentially associated with air pollution and other factors, whereas our estimate is based on heat mortality only. Finally, we have focused on the harmful health effects of an extreme heatwave event in this work, although the UHI effect is present throughout the year, and most pronounced at night. It is possible therefore that there is a ‘protective’ effect on health due to increased urban temperatures in city centres during cold spells, and this requires further research. Similar modelling techniques can be employed to explore this further in future work.

The results from this work have practical implications for health impact assessment for heat-related health effects in urban areas for the UK, and elsewhere. Improvements can be made to the methodology for health impact assessments by using temperature data which accurately reflects temperature exposure for the population. In addition, high resolution urban temperature simulations can be used as input to building energy models to help quantify building overheating in urban areas during heatwaves.

## Additional file

Additional file 1:
**Peer review reports.** (PDF 105 kb)
