# Supplementary material for: Attribution of mortality to the urban heat island during heatwaves in the West Midlands, UK
Source: Environ Health. 2016 Mar 8;15(Suppl 1):27. doi: 10.1186/s12940-016-0100-9 (PMC4895726; doi:10.1186/s12940-016-0100-9)
Supplement: Additional file 1: — Peer review reports. (PDF 105 kb) [file 12940_2016_100_MOESM1_ESM.pdf]

Reviewer 1

General Comments

This paper provides an interesting contribution on the contribution of the UHI impact to urban mortality during heat waves. The layout is generally clear and I have just a few minor comments. There may be some scope for slightly shortening the paper; in a few places I felt that some repetition occurred and ensuring that the assumptions used are stated early in the paper.

Specific Comments

Pg 3 2nd paragraph – I would recommend more precisely defining the heat island of interest (it is the air temperature heat island in the urban canopy layer – or canopy layer uhi) and providing more context for the 10 C value quoted (maximum expected nocturnal heat island between the most built up part of the city and the rural surroundings under optimum weather conditions – clear and calm).

Pg 4 3rd paragraph – are UHI modified by change in future urbanization?

Pg 5 1st paragraph – Does the model evaluation include stations that are explicitly urban? Or just stations from rural surroundings ?
What time was the maximum 7 C heat island observed?

Pg 5 2nd paragraph – are the moisture conditions for the rural 'dryland cropland and pasture' a fixed parameter in the model or is it set in the initialization? Are these the same values for both the urban and rural runs? UHI magnitude can be sensitive to the rural conditions (especially how moist or dry they are) – for a specific heat wave, the urban-rural difference may be larger for dry rural conditions (at night when they can cool more because of their low thermal admittance – eg. Runnalls and Oke 2000) – can you comment on the setup of the simulation as it represents rural conditions?

Pg 5 last paragraph – could be worth mentioning that there is work in which urban parameterizations are added to global scale simulations e.g. work by Oleson and colleagues; the paper by McCarthy et al. 2010 also describe how the UHI may change in future cities (although I think that work does not include urban development)

Pg 7 1st paragraph – last line –suggest "5.31 million in 2003).

Pg 8 2nd paragraph, 5th last line suggest "…generally a smaller diurnal temperature range in urban areas." Rural areas "do not generally store heat" – seems a bit of an overstatement – the release of heat is faster in rural areas compared to urban, and this is sensitive to their moisture content.

Pg 11 1st paragraph – "was on average 3.2 C" – over what period is this? A day? Longer?

Pg 11 last paragraph – my interpretation here is that the estimates are conservative – is this correct? Perhaps comment on how conservative (or not) the estimates are?

Pg 12 2nd paragraph – 2nd last sentence – Presumably the estimates also assume no change in UHI either because of weather, rural conditions or urban development.

Pg 13 2nd paragraph – city size has some impact on heat island magnitude but the main control is the degree of development as determined by physical measures such as H/W ratio, replacement of natural surfaces with built materials etc.

Pg 13 last paragraph, last sentence – The position of vulnerable populations – here variations in outdoor conditions are determined – but in reality the location of vulnerable populations must consider indoor conditions that are quite site specific: building stock quality, operation (or not) of windows, insulation, location of inhabitants (e.g. on upper floors?), their health status etc.

Pg 14 "We also assume that the urban density…" – I think it would be worth stating this earlier in the paper.

Minor / Editorial/typographical comments

Pg 4 3rd paragraph, second last sentence is awkward: suggest "…such as we might expect in future decades under climate change scenarios."

Pg13 2nd paragraph – 2nd sentence – awkward wording

Pg 16 – Figure 1 caption suggest "… and is 1 km^2 in the central domain…"

References

McCarthy, M. P., M. J. Best, and R. A. Betts (2010), Climate change in cities due to global warming and urban effects, Geophys. Res. Lett., 37, L09705, doi:10.1029/2010GL042845.

Reviewer 2

Review EH
The contribution of the Urban Heat Island to heat related mortality during the 2003 heatwave and for projected future climate in the West Midlands, UK

Conflicts of interest:
I have no real or potential personal or financial conflict of interest related to the review of this manuscript.

Recommendation: major revision

Comments:
General Comments

The authors conducted a health impact assessment of an event of heat wave (as a case of Aug 2003) using UHI modelling data and UKCP09 future projection scenarios, assuming no acclimatization or adaptation in the case of West Midland. The concept of UHI intensity comparing urban and rural areas is interesting, but some challenging assumptions made main messages vague and sceptical. My largest concerns are comparisons in Table2: for example, assuming rural surface, but calculating health impact of urban population from these attenuated temperature due to rural surface; generally, UHI modelled temperature will be hardly aggregated to regional mean; population weighting at this level (1km modelled temperature) will bring other complicated uncertainties in future projection. Some errors in basic epidemiological terms or concepts need to be addressed appropriately for Journal of EH. Specific comments are below.

Major Comments

1. In Abstract: Objectives are unclear. Is this to quantify the contribution of the UHI to heat related mortality in the West Midlands and/or to estimate future health burdens? It would be clearer if the readers have some guides to follow objectives/methods/results/conclusion (either with or without headings).
2. In Abstract: the first sentence (people who live in cities are particularly vulnerable to adverse health impacts during heatwaves due to the UHI effect) is not based on the evidence (they may have adapted/acclimatized in their local climate). I suggest to rephrase based on clear evidence. For example, vulnerability to heat in urban area is anticipated due to observed elevated outdoor temperature in the urban area.
3. In Abstract: the authors say “geographical mean under-estimates the population exposure to temperatures (in the urban area) by around 1d/C”. But it would be over-estimated in rural area (although most of the deaths might have occurred in urban area). Explain what is the point to average UHI gridded modelled temperature to region by geographical or population-weighted mean? Generally, spatially-disaggregated modelling (temperature or air pollution) is to reflect local environment at that geographical level (in this case, 1km grid). Most of the studies try to keep the similar geographical level rather than aggregating to region, if possible. In this case, the authors have 1km or even 100m grid resolution of population data and could proceed HIA at that level, then sum up the estimated deaths to region level.
4. In Background: the 1st sentence. References 1-5 are referred to future health impacts using climate change projection. Add Knowlton et al. 2007 in American Journal of Public Health which accounted for modelled acclimatization from analogue cities.
5. In Background: the 3rd paragraph, definition of the UHI intensity (difference in temperature between urban and rural locations). Due to implausible assumption (that estimating health

impact of large population in urban area using attenuated rural temperature, assuming a large population would live in the setting of rural condition such as "dryland cropland and pasture"?), it would be better to see the difference between modelled temperature (called as `urban run' by authors) and regional mean of modelled temperature or between modelled temperature (`urban run') and hypothetical modelled temperature replaced with more region-representative land-surface (with reasonable population reside).

6. In Background: the 5th paragraph. The authors say "This means that health impact assessment based on climate projections may underestimate the potential health impacts of …" However, possibility of overestimation is generally more concerned due to assumptions of no-adaptation. And people will reside in not only urban area, but also rural area. I suggest the authors not to reveal "underestimate" if their model doesn't take into account for adaptation.
7. In the 6th paragraph. "population exposure as accurately as possible". "accurate" will probably bring other unnecessarily concerns about indoor-temperature etc. Please be careful here.
8. In the 7th paragraph. "to our knowledge, this study is the first to attempt…." See Impact of London's Urban Heat Island on Heat-related Mortality (Milojevic et al. 2011 in Epidemiology 22(1) S182-183), which keeps 1km grid to estimate impact and sums up by UHI zones.
9. In the 7th paragraph. "the use of high resolution simulation…. And allows for population weighting for temperature exposure." Population-weighted temperature could be implemented by monitored temperature as well (Armstrong JECH paper did actually). I think it's better to focus that the merit of high resolution simulation is to model UHI. (see other comments related with population-weighting at this spatial scale, as well)
10. The 2nd last sentence in the Background: "We calculate <u>the likely impacts</u> on health of an increase in temperature such as we might expect in the future under climate change scenarios for future decades in addition to the UHI intensity". I think it is <u>the most over-estimated</u> impact due to UHI, climate change, population with no adaptation). If you present these estimated figures, probably it's better to show the author's notion about this in Discussion.
11. Temperature modelling. Explain how WRF model output was used to simulate UHI. Hourly temperature was averaged to daily average? – Hajat's temperature-mortality relationship (JECH) is based on daily measure, thus it should be coincided. Is there any specific name for UHI model? I know the original method paper was cited here, but briefly it should be explained here for the reader to follow the process easily.
12. Temperature modelling: the 1st paragraph. the UHI intensity should be defined here, in the Method section.
13. Climate Projections: in the 2nd paragraph. Is there any detailed description how UKCP09 and UHI modelling was linked? IFfso, cite the publication or describe briefly. Currently, it is not easy to follow what is input for and output from each model.
14. Climate Projections: in the 2nd paragraph. 2080s seems too far to estimate health impact (though reference [1] paper tried). Uncertainties in population structure and adaptation/acclimatization as well as temperature-mortality relationship makes estimates unrealistic, if the target future period gets too far. Though climate modellers have little interests if it's limited to next couple of decades, but from the health perspective, I suggest to keep the 2020s and the 2050s only, unless the authors have specific reason to include the 2080s.
15. Demographic data: Is population-weighted temperature necessary in such a local level modelled temperature (1km grid)? Because the author use the same population weights to estimate health impacts in the 2020s and 2050s, they need to assume population density at 1km-level resolution remains consistent over a couple of decades, which is very unlikely. Why do you need to aggregate 1km grid temperature to regional average by population weight? It would be less information-loss if you could keep 1km grid temperature and population density to compute health impacts by grid, then aggregate heat-attributable deaths to region.
16. In health impact assessment methodology: applied threshold (17.7dC) is described. In addition, region-specific exponentiated beta (RR) should be explained with 95%CI. Currently only 1.02

(point estimate?). Though the author referred Hajat et al.'s paper, it should be explained briefly how these threshold and beta were derived (especially, what were adjusted for).

17. In Health impact assessment methodology: the 2nd paragraph. "RR of mortality for heat in the West Midlands at the threshold value of 17.7d/C". Do you mean above the threshold? Also, description of lag effects seems to be confusing. Why temperature on the final day needs to be taken as that of the previous day? If you have simulated temperature for 1st -10th August (till midnight), then you should be able to estimate health impact for 2nd -10th with lag 0-1 days.
18. In Health impact assessment methodology: the last sentence. "exposure response relationship as for the 2003 analysis"? Temperature (heat)-mortality relationship in Hajat et al. was based on 1993-2006 as described in the previous paragraph. Keep consistent.
19. In Results: please report only the finding from this work. Some parts are extensions from the Methods (for example, "although we use simulated temperature data, observations from fixed …" needs to be replaced in the Methods section appropriately. It's confusing)
20. In population-weighted mean temperature in the West Midland: see the related comment above. I don't see point to use 100m grid population data here. This brings more complication (assumption to remain in future etc.) and indoor temperature might be more critical aspect to improve exposure measure. NPD database includes daytime population, night time population, and vulnerable groups (pupils, care-home residents etc), which might be more relevant to close-investigation.
21. Figure 2: currently, night-time hours map only. Although night-time temperature may show apparent UHI effects, whole-period average map (day and night) is more relevant information to support methodology of this HIA as the authors used daily mean temperature (is it right? It was not explained). So, I suggest to have both daily mean and night-time mean temperature maps.
22. In population-weighted mean temperature: the 1st sentence in the 4th paragraph. Please explain, what is the point to take geographical or population-weighted mean temperature of 1km grid UHI temperature. (see the similar comment above)
23. "This could have an important effect on the results of health impact assessments, particularly over extended periods of time, as errors accumulate." - Be cautious to use the term "error". "Error" has to be defined here with corresponding "correct" values (as reference).
24. In Health Impact Assessment - heat related mortality during the heatwave: the first 2 paragraphs should be in methods.
25. "In other words, these calculations indicate that the UHI effect and the increased density of the population residing in city centres may account for 54% of the total heat related mortality." I am not sure what this 54% really mean. As commented above, health impacts of urban population were estimated by the rural attenuated temperature (Can we really assume large population live in such rural condition?)
26. Table 2: I suggest to reconsider the reasonable comparison with notions on involved assumptions under the table. Probably this would be connected with the Objectives, once the authors clarify them. A comparison something to make clear about UHI contribution (attribution) would be suitable for the (current) title of this paper.
27. In Health impact assessment - mortality for projected changes in climate: Most of parts are not relevant for the Results section. The results from this work seem to be from the last 2 lines in p11 to the 2nd last sentence before Table3 only. In the last sentence before Table3, "RR relationship" is meant as heat-mortality relationship? "as for 2003" should be "as for 1993-2006".
28. Tabl3: delete 2080s (see the related comment above). All assumptions should be noted under the table (for 2050s, the UK population was assumed etc.)
29. In Discussion and Conclusions: the last paragraph on p13. "when considering that temperature exposure in urban areas may be a few degrees higher than existing predictions based on observations taken outside of cities". This is good finding from this study.

30. The 1st paragraph on p14 needs to be extended more as limitations of this study. The author says "population adaptation" is difficult to predict, quantify and account for in a HIA, but Knowlton et al. tried to model adaptation and Kinney et al. (reference 5) discusses a few approaches to handle this as well as limitations in future projections. It would be good if the discussion here could be expanded more to address some of Kinney et al.'s points, such as mortality displacement.
31. The authors say "the UHI effect is present throughout the year". Can they discuss what this work implies possible attenuation of cold-related mortality?
32. If possible, can the authors add a figure to show land surface of urban and rural (or even sub-categories)? It would be useful to give apparent information about what is the settings for urban-model run and rural-model run.

<u>Minor Comments</u>

33. In Abstract: 2nd paragraph. "The contribution of the UHI" could be more specific, such as "attribution of the elevated temperature due to the UHI"? (if there are room for word limits)
34. In Abstract: what do you mean by "spatial analysis of temperatures"? Probably "spatially-disaggregated temperature" suites better in this context?
35. In Methods: Temperature modelling. "gridded 2 meter air temperature" -> temperature at the height of 2m? Sub-x title in Figure2 should be changed accordingly.
36. Figure 1: please add the scale of distance for each map. If this study estimates health impact in the West Midland region (rather than right-hand detailed map extent), then add region boundaries in Figure1-3.
37. In The impact of the UHI on heat-related mortality: the first two sentences are not relevant for Result section. In the 2nd paragraph, the UHI intensity (the urban simulation) is different definition from the previous one (difference between urban run and rural run). Be consistent.
38. "Vulnerable populations in urban centres" - NPD database could be used to identify some of the vulnerable groups (pupils, care home residents, etc.). Probably extend this in Discussion part?

That is all.
